# Association between EAT-Lancet diet adherence and cancer incidence/mortality: a systematic review and meta-analysis

**DOI:** 10.3389/fonc.2026.1823812

**Published:** 2026-06-01

**Authors:** Xinhe Li, Jingqi Chen, Zhanqi Sun, Minyuan Ni, Minmin Fu, Yunshi Tang

**Affiliations:** 1 The Second School of Clinical Medicine, Zhejiang Chinese Medical University, Hangzhou, Zhejiang, China; 2 The First Affiliated Hospital of Zhejiang Chinese Medical University (Zhejiang Provincial Hospital of Chinese Medicine), Hangzhou, Zhejiang, China; 3 Health Science Center, Ningbo University, Ningbo, Zhejiang, China; 4 School of Life Sciences, Zhejiang Chinese Medical University, Hangzhou, Zhejiang, China; 5 Department of Clinical Nutrition, The First People’s Hospital of Pinghu, Jiaxing, Zhejiang, China

**Keywords:** cancer, EAT-Lancet diet, incidence, lung cancer, meta-analysis, mortality

## Abstract

**Background:**

Cancer is a major threat to public health around the world. Diet is a key factor that we can change to help prevent it. This study explores the association between adherence to the EAT-Lancet diet and cancer incidence and mortality.

**Methods:**

A systematic search was conducted across PubMed, Web of Science, Embase, and the Cochrane Library for cohort studies published from January 2019 to September 2025. In the primary analyses, we pooled adjusted hazard ratios (HRs) with 95% confidence intervals (95% CIs) for the association between EAT-Lancet diet adherence scores and cancer outcomes, prioritizing linear score-increment estimates and using categorical adherence contrasts only when linear estimates were unavailable. Subgroup analyses were performed by cancer type and adherence level; adherence-level subgroup analyses used categorical contrasts reported in the original studies. Sensitivity analysis and publication-bias assessment were conducted.

**Results:**

A total of 15 cohort studies were included. In the primary analyses, higher EAT-Lancet diet adherence scores were linearly associated with lower overall cancer incidence and overall cancer mortality (incidence: HR = 0.90, 95% CI: 0.84-0.95, P<0.001; mortality: HR = 0.92, 95% CI: 0.90-0.95, P<0.001). The clearest site-specific associations were observed for lung cancer incidence (HR = 0.93, 95% CI: 0.90-0.95, P<0.001) and lung cancer mortality (HR = 0.94, 95% CI: 0.90-0.97, P<0.001). No statistically significant associations were observed for breast, prostate, or colorectal cancer incidence (P>0.05).

**Conclusion:**

Higher EAT-Lancet diet adherence scores may be associated with lower overall cancer incidence and mortality, with the clearest association observed for lung cancer. These findings should be interpreted cautiously because of heterogeneity, residual confounding, and differences in adherence assessment across cohorts.

**Systematic review registration:**

https://www.crd.york.ac.uk/prospero/, identifier CRD420251141170.

## Introduction

According to the special topic analysis on cancer from the global burden of disease study, there were 18.5 million new cancer cases worldwide in 2023 (excluding non-melanoma skin cancer) and 10.4 million cancer deaths (1), making cancer a major global public health threat. Thus, researching its prevention and control is essential. Cancer progression is influenced by a combination of non-modifiable internal factors and modifiable external factors. Internal factors comprise genes and ethnicity, whereas external factors include, but are not limited to, environmental factors, diet, smoking, and access to medical services (2). Among these, diet is a key modifiable factor, and accumulating evidence indicates that diet and nutrition contribute to 20–25% of the global cancer burden (3, 4). For instance, some studies have indicated that a fiber-rich healthy dietary pattern (e.g., prudent diet) is associated with a lower risk of colorectal cancer, and that a Mediterranean diet supplemented with extra-virgin olive oil reduces breast cancer risk (5).

The EAT-Lancet diet, proposed in 2019, aims to balance human health and environmental sustainability (6). It has been associated with favorable health outcomes, including lower risks of diabetes, cardiovascular disease mortality, and all-cause mortality (7). Previous meta-analyses on this association showed that the EAT-Lancet diet was associated with lower cancer incidence but had notable limitations: they included a small number of studies. Moreover, they did not conduct subgroup analyses based on cancer types or dietary adherence levels (8).

To address these limitations, this study aims to review relevant cohort studies to assess the relationship between EAT-Lancet diet adherence and cancer incidence and mortality. The ultimate goal is to provide some references for health promotion.

## Materials and methods

### Study design

This study is a meta-analysis, aiming to assess the association between adherence to the EAT-Lancet diet and the risk of cancer incidence and mortality. The study was conducted in accordance with the Preferred Reporting Items for Systematic Reviews and Meta-Analyses (PRISMA) statement. It was prospectively registered in the PROSPERO database with the registration number CRD420251141170.

### Search strategy

To systematically search for studies, two researchers conducted a systematic search across four core electronic databases: PubMed, Web of Science, Embase, and the Cochrane Library. The search period was set from January 2019 to September 2025, with no language restrictions during the search. When constructing the search strategy, we combined MeSH terms and free terms, and applied boolean logic. The search string was as follows: ((((EAT-Lancet diet) OR (planetary health diet)) OR (EAT-Lancet score)) OR (planetary health diet index (PHDI))) AND ((((((((“neoplasms”[Mesh]) OR (neoplasms)) OR (neoplasia)) OR (neoplasm)) OR (tumor)) OR (cancer)) OR (cancers)) OR (malignant neoplasm)). To avoid missing eligible studies, we also screened the reference lists of included articles and relevant reviews. Grey literature, dissertations, non-peer-reviewed reports, conference abstracts, letters, and records without full-text data were not included because this meta-analysis required full cohort reports with extractable adjusted effect estimates.

### Inclusion and exclusion criteria

We developed the inclusion criteria based on the PECOS framework, which are as follows: participants (P) were adults aged >=18 years; exposure (E) was adherence to the EAT-Lancet diet or EAT-Lancet diet score; comparison (C) was a linear increment in adherence score or intergroup contrast across different adherence levels; outcomes (O) were cancer incidence or cancer mortality; and study design (S) was a cohort study.

Meanwhile, we defined the exclusion criteria. A study was excluded if it met any of the following conditions: full text or key data were unavailable; the publication type was an abstract, conference proceeding, letter, review, or other non-original report; the exposure or outcome did not match the PECOS criteria; or the data overlapped with another report from the same cohort. For overlapping or updated cohorts, only the report with longer follow-up, more complete outcome information, or more comprehensive adjusted estimates was retained.

### Definition of the EAT-Lancet diet

The EAT-Lancet diet is a planetary health-oriented dietary pattern that balances human health and environmental sustainability. Its core recommendations include: at least 250 grams per day of whole grains, at least 300 grams per day of vegetables, at least 200 grams per day of fruits, moderate intake of legumes and nuts, less than 100 grams per week of red meat, and less than 25 grams per day of added sugars (9).

Across included cohorts, adherence was operationalized using the original or adapted EAT-Lancet diet scoring system reported by each study. For the primary analyses, linear score-increment estimates were preferred. When linear estimates were unavailable, categorical adherence contrasts were used. Due to differences in the scoring systems and increment units adopted by different cohorts, uniform conversion was not feasible. Therefore, the increment units as defined in each original study were retained. Categorical cutoffs, such as tertiles, quartiles or quintiles, were used for adherence-level subgroup analyses. Details of scoring systems, cutoffs, and dietary assessment tools are provided in **Supplementary Table 1**.

### Data extraction and quality assessment

Data extraction was independently completed by two researchers using a pre-designed form. If there were discrepancies between the two researchers during the extraction process, they first resolved them through discussion; if consensus still could not be reached after discussion, a third researcher was consulted. The extracted information included: study basic information (authors, publication year, country of origin), cohort information (cohort name, recruitment period, follow-up time, sample size, age), exposure details (assessment methods and evaluation tools for EAT-Lancet diet adherence), and outcome data (the risk of cancer incidence and mortality). For studies with missing data, we made an attempt to contact the authors via email to obtain supplementary information.

For quality assessment, given that all included studies were cohort studies, the Newcastle-Ottawa Scale (NOS) was used, focusing on three domains: representativeness of the cohort, measurement of exposure (e.g., use of validated dietary questionnaires to assess EAT-Lancet diet adherence), and follow-up (requiring the median follow-up time ≥5 years and the loss to follow-up rate <20%). Quality assessment was also conducted independently by two researchers; if discrepancies occurred, they were resolved by consulting a third researcher.

### Statistical analysis

Statistical analyses were performed using RevMan 5.4 and Stata software. In the primary analysis, one adjusted HR was selected for each eligible study outcome. Linear score-increment estimates were used preferentially. If a linear estimate was unavailable, the most fully adjusted categorical adherence comparisons was used. When both estimate types were reported for the same outcome, the linear estimate was included in the primary analysis. The natural logarithm of each selected HR and its standard error were pooled using a generic inverse-variance random-effects model. In the subgroup analysis by adherence level, studies were grouped according to categorical adherence comparisons reported in the original articles. Heterogeneity was evaluated using the chi-square test, with the I^2^ statistic representing the degree of heterogeneity. The specific criteria were: I^2^ <25% for low heterogeneity, 25% ≤ I^2^ <50% for moderate heterogeneity, and I^2^ ≥50% for high heterogeneity.

Given the inherent heterogeneity among included studies in population characteristics, cancer type, dietary assessment tools, EAT-Lancet scoring systems, and dietary habits, a random-effects model was selected as the primary model. Subgroup analyses were conducted by cancer type and adherence level to explore potential sources of heterogeneity. Leave-one-out sensitivity analyses were performed for the overall cancer incidence and mortality analyses to assess robustness. These sensitivity analyses used the same estimate set as the primary analyses, in which linear score-increment estimates were prioritized and categorical contrasts were used only when linear estimates were unavailable. Publication bias was explored using funnel plots only. Egger’s test was not performed because it is statistically unstable when fewer than 10 studies are included. All statistical tests were two-tailed, and P<0.05 was considered statistically significant.

## Results

### Study selection process

**Figure 1:**
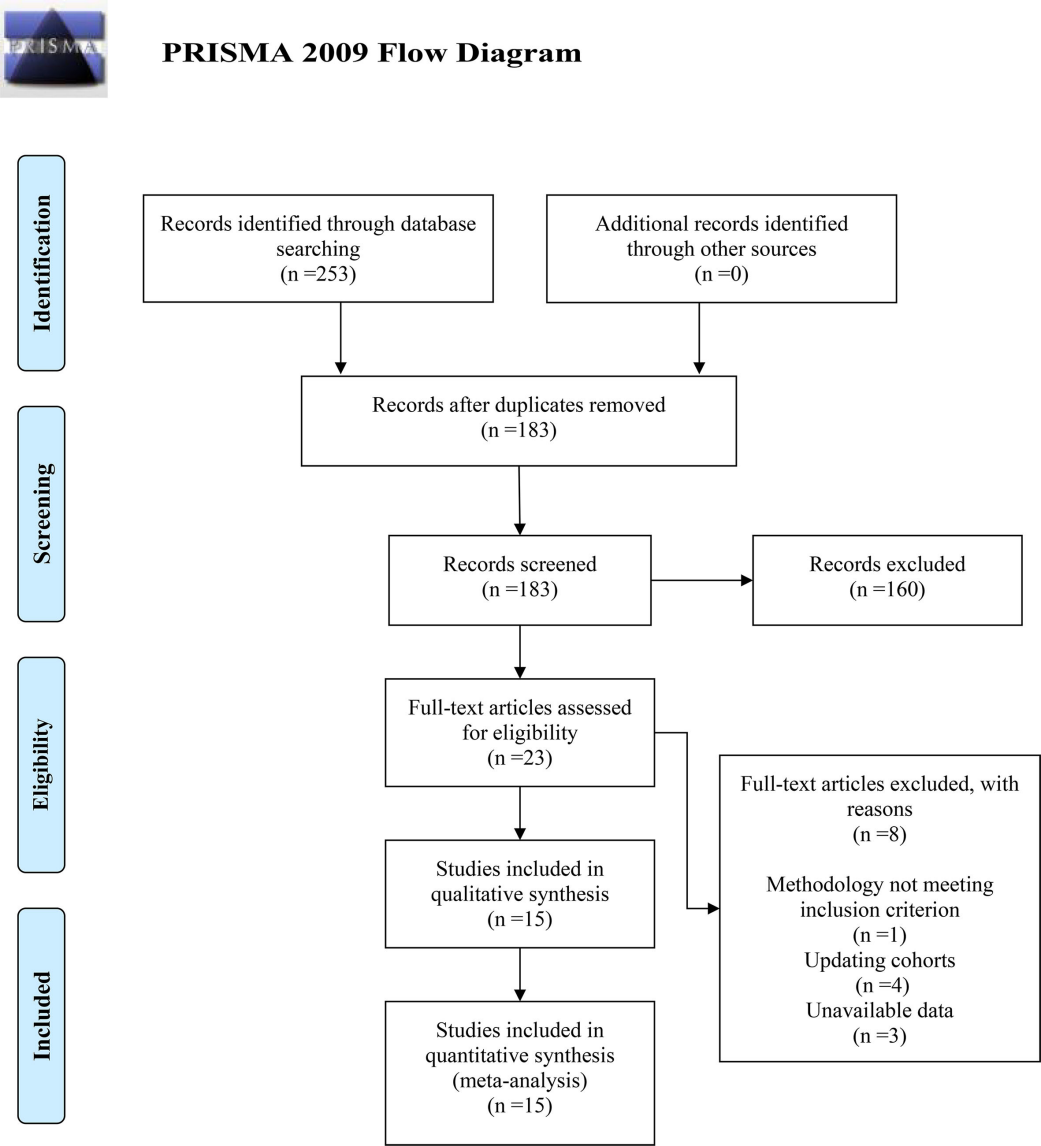
Flow diagram of the selection process.

A systematic literature search was conducted in PubMed, Web of Science, Embase, and the Cochrane Library using predefined search terms. The search initially identified 253 records; no additional records were obtained from other sources. After removing duplicate records, 183 records remained for screening. By reviewing titles and abstracts, 160 records were excluded. For the remaining 23 records, full-text reviews were performed, and 8 more studies were excluded: 1 with methodology not meeting the inclusion criteria, 4 overlapping or updated cohort reports, and 3 with unavailable data. Finally, 15 cohort studies were included in the meta-analysis (10) (11–24), and the detailed selection process is presented in **Figure 1**.

### Basic characteristics of included studies

All 15 included studies were cohort studies, published between 2022 and 2025, and conducted in countries such as the United Kingdom, the United States, Italy, Spain, Sweden, and Singapore. These studies used several large cohorts, including the UK Biobank, PLCO, NutriNet-Sante, ORDET, COSM/SMC, NHANES, ENRICA, MDCS, NHS/HPFS, BWHS, and SCHS. Recruitment periods ranged from 1976 to 2021, and follow-up durations ranged from 8.1 to 32.17 years. The cancer outcomes investigated included overall cancer, breast cancer, colorectal cancer, prostate cancer, lung cancer, renal cancer, oral and pharyngeal cancer, laryngeal cancer, and cancer mortality. Detailed characteristics are summarized in **Table 1**. Further details regarding age at recruitment, EAT-Lancet diet adherence grouping criteria, scoring systems, and dietary assessment tools are summarized in **Supplementary Table 1**.

**Table 1:** Characteristics of studies included in the meta-analysis.

Author	Year	Cohort name	Recruitment period	Sample size, N	EAT–lancet diet adherence assessment	Dietary assessment tool	Median follow–up time (years)	Outcome indicator
Karavasiloglou, N.	2023	UK Biobank	2006–2010	473,836	EAT–Lancet reference diet score	UK Biobank touchscreen questionnaire	10.49	Breast cancer incidence Colorectal cancer incidence Prostate cancer incidence
Liu, F.	2024	UK Biobank	2006–2010	175,214	ELD score	Oxford WebQ	9.47	Lung cancer incidence Lung cancer mortality
Ren, X.	2023	PLCO	1993–2001	98,415	ELD score	DHQ	8.82	Colorectal cancer incidence
Xiao, Y.	2023	PLCO	1993–2001	101,755	ELD score	DHQ	8.82	Lung cancer incidence Lung cancer mortality
Wei, Q.	2025	PLCO	1993–2001	101,755	ELD score	DHQ	8.84	Renal cancer incidence
Ren, X.	2024	PLCO	1993–2001	101,755	ELD score	DHQ	8.84	Oral and pharyngeal cancer incidence laryngeal cancer incidence
Berthy, F.	2022	NutriNet–Sante	2009–2021	62,382	ELD–I	Nonconsecutive 24–hour dietary record	8.1	Colorectal cancer incidence Lung cancer incidence Breast cancer incidence Prostate cancer incidence
Quartiroli, M.	2024	ORDET	1987–1992	9144	EAT-Lancet score	FFQ	22.6	Breast cancer incidence
Pitt, S.	2024	COSM, SMC	1997	68,175	EAI	FFQ	22	Cancer mortality
Han, S.	2025	NHANES	2005–2018	30,521	Planetary Health Diet Index for the United States	24–hour dietary recalls	8.5	Cancer mortality
Aznar de la Riera, M. d. C.	2025	ENRICA	2008–2010	11,448	PHDI	DH–ENRICA	14.4	Cancer mortality
Stubbendorff, A.	2022	MDCS	1991–1996	22,421	ELD–I	Modified diet history method	20	Cancer mortality
Bui, L. P.	2024	NHS1, NHS2, HPFS	NHS1:1976, NHS2: 1989, HPFS: 1986	203,405	PHDI	FFQ	NHS1: 27.5, NHS2:32.17, HPFS: 30.92	Cancer mortality
Shan, Y.	2025	BWHS	1995	33,824	PHDI	FFQ	18	Cancer mortality
Ye, Y.–X.	2023	SCHS	1993–1998	57,078	PHD score	FFQ	23.4	Cancer mortality

PLCO, Prostate, Lung, Colorectal and Ovarian Cancer Screening Trial; ORDET, Hormones and Diet in the Etiology of Breast Cancer; COSM, Cohort of Swedish Men; SMC, Swedish Mammography Cohort; NHANES, National Health and Nutrition Examination Survey; ENRICA, Estudio de Nutricion y Cancer (Spain); MDCS, Malmo Diet and Cancer Study; NHS1, Nurses’ Health Study I; NHS2, Nurses’ Health Study II; HPFS, Health Professionals Follow-up Study; BWHS, Black Women’s Health Study; SCHS, Singapore Chinese Health Study; PHD, Planetary Health Diet; PHDI, Planetary Health Diet Index; ELD-I, EAT-Lancet Diet-Index; ELD, EAT-Lancet Diet; EAI, EAT-Lancet Adherence Index; DHQ, Dietary History Questionnaire; FFQ, Food Frequency Questionnaire; DH-ENRICA, Dietary History in Estudio de Nutricion y Cancer (Spain).

### Quality assessment of included studies

The quality of the 15 cohort studies was evaluated using the Newcastle-Ottawa Scale (NOS), which assesses selection, comparability, and outcome domains with a maximum score of 9 points. All included studies scored between 7 and 9 points and were classified as moderate to high quality. This suggests that the included evidence was generally reliable, but quality appraisal did not eliminate important methodological concerns, including self-reported dietary assessment, differences in covariate adjustment, and residual confounding. The detailed quality assessment results are shown in **Supplementary Table 2**.

### Cancer incidence-related results

**Figure 2:**
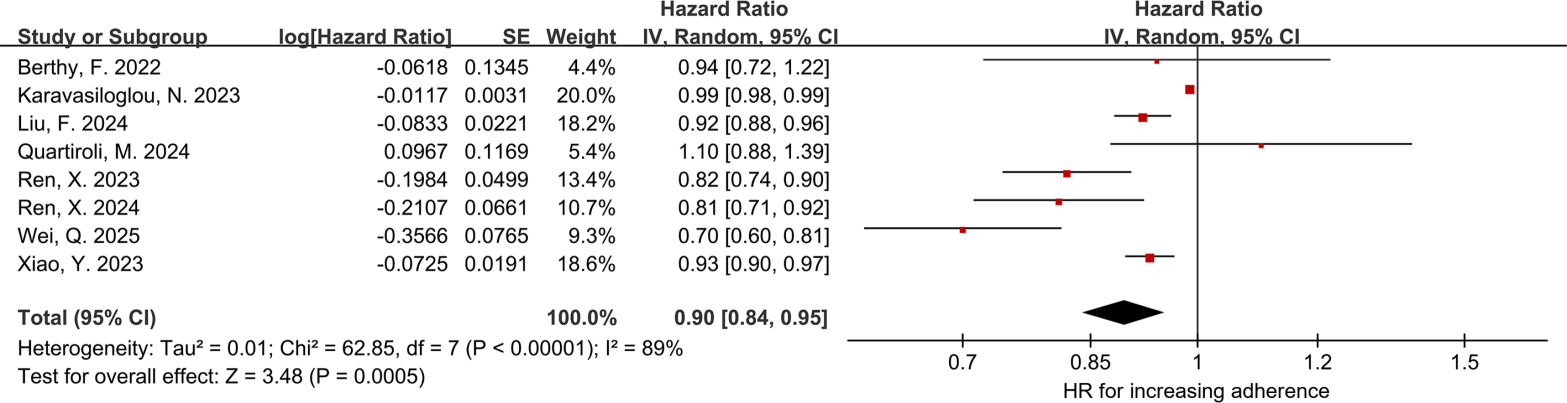
Forest plot of EAT-Lancet diet and incidence of cancer.

Eight studies reported data on cancer incidence. For the primary analysis, five estimates were linear score-increment estimates (Berthy, Karavasiloglou, Liu, Ren 2024, and Xiao), and three were categorical adherence contrasts used because linear estimates were unavailable (Quartiroli, Ren 2023, and Wei). As shown in **Figure 2**, the primary analysis showed higher EAT-Lancet diet adherence scores were associated with lower overall cancer incidence (HR = 0.90, 95% CI = 0.84-0.95, P<0.001). Heterogeneity was substantial (I^2^=89%). Leave-one-out sensitivity analysis demonstrated that the direction and magnitude of the pooled association remained consistent after sequentially omitting each individual study, indicating the robustness of our primary findings (**Supplementary Figure 1**). Visual inspection of funnel plots revealed a roughly symmetrical distribution, suggesting no significant publication bias in the present meta-analysis (**Supplementary Figure 2**).

**Figure 3:**
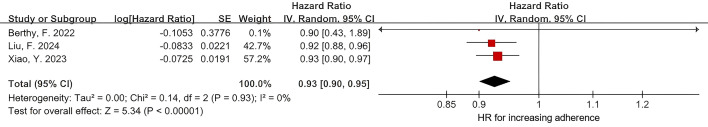
Forest plot of EAT-Lancet diet and incidence of lung cancer.

Subgroup analysis of cancer incidence was first conducted by cancer type. Three study estimates reported lung cancer incidence using linear adherence-score estimates, and the pooled result showed that higher EAT-Lancet diet adherence scores were associated with lower lung cancer incidence (HR = 0.93, 95% CI = 0.90-0.95, P<0.001), with no significant heterogeneity (I^2^=0%), as shown in **Figure 3**. In addition, 3 studies reported breast cancer incidence (HR = 0.99, P = 0.380), 2 studies reported prostate cancer incidence (HR = 1.08, P = 0.630), and 3 studies reported colorectal cancer incidence (HR = 0.90, P = 0.190). No statistically significant associations were observed for these cancer types; further details are provided in **Table 2**. Adherence-level subgroup analysis was based on categorical contrasts reported in the original studies. For high adherence versus low adherence, no significant difference was detected for overall cancer (HR = 0.84, P = 0.120), breast cancer (HR = 0.96, P = 0.460), prostate cancer (HR = 1.08, P = 0.650), or colorectal cancer (HR = 0.88, 95% CI: 0.78-1.00, P = 0.060). For moderate adherence versus low adherence, no significant difference was found for overall cancer (HR = 0.90, P = 0.280), breast cancer (HR = 0.98, P = 0.550), prostate cancer (HR = 1.19, P = 0.410), or colorectal cancer (HR = 0.99, P = 0.830).

**Table 2:** Subgroup analysis of cancer incidence based on cancer types and EAT-Lancet diet adherence.

Subgroup	No. of studies	HR	95% CI	P	I2 (%)
Breast cancer	3	0.99	(0.98, 1.00)	0.380	0
Prostate cancer	2	1.08	(0.80, 1.44)	0.630	47
Colorectal cancer	3	0.90	(0.76, 1.06)	0.190	84
High dietary adherence					
Overall cancer	3	0.84	(0.67, 1.05)	0.120	86
Breast cancer	3	0.96	(0.89, 1.04)	0.460	0
Prostate cancer	2	1.08	(0.76, 1.54)	0.650	80
Colorectal cancer	2	0.88	(0.78, 1.00)	0.060	0
Moderate dietary adherence					
Overall cancer	3	0.90	(0.75, 1.09)	0.280	79
Breast cancer	3	0.98	(0.94, 1.03)	0.550	0
Prostate cancer	2	1.19	(0.79, 1.80)	0.410	86
Colorectal cancer	2	0.99	(0.94, 1.05)	0.830	0

No., number; HR, hazard ratio; CI, confidence interval.

### Cancer mortality-related results

**Figure 4:**
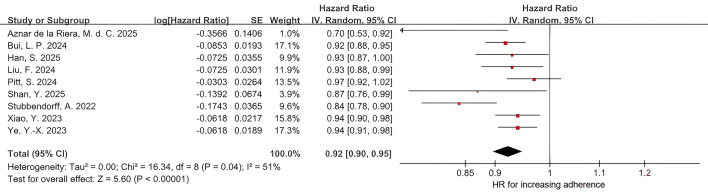
Forest plot of EAT-Lancet diet and mortality of cancer.

**Figure 5:**
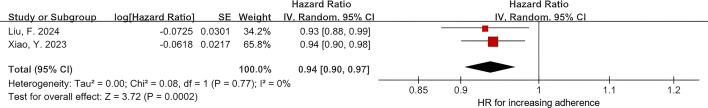
Forest plot of EAT-Lancet diet and mortality of lung cancer.

Nine studies reported data on overall cancer mortality (**Figure 4**). For the primary analysis, five estimates were linear score-increment estimates (Bui, Han, Liu, Pitt, and Xiao), and four were categorical adherence contrasts used when linear estimates were unavailable (Aznar de la Riera, Shan, Stubbendorff, and Ye). The primary analysis showed that higher EAT-Lancet diet adherence scores were associated with lower overall cancer mortality (HR = 0.92, 95% CI: 0.90-0.95, P<0.001), with moderate heterogeneity (I^2^=51%). Leave-one-out sensitivity analysis showed that our pooled estimate was highly robust, as exclusion of any single study did not materially change the direction or magnitude of the effect (**Supplementary Figure 3**). Funnel plots were generally symmetrical, with no visual evidence of publication bias (**Supplementary Figure 4**). Two studies reported lung cancer mortality (**Figure 5**), and higher EAT-Lancet diet adherence scores were associated with lower lung cancer mortality (HR = 0.94, 95% CI: 0.90-0.97, P<0.001), with no significant heterogeneity (I^2^=0%). Both lung cancer mortality estimates were linear score-increment estimates.

## Discussion

This meta-analysis suggests that higher EAT-Lancet diet adherence scores are associated with lower overall cancer incidence and mortality in adults, with the most consistent site-specific association observed for lung cancer. No statistically significant association was observed for breast, prostate, or colorectal cancer incidence. These findings extend previous syntheses by incorporating updated cohort evidence and by conducting subgroup analyses by cancer type and adherence.

Modern diets vary by region, but many populations are experiencing a shift toward higher consumption of red and processed meats, refined grains, added sugars, saturated fats, and ultra-processed foods, together with lower intake of fruits, vegetables, whole grains, and dietary fiber (5) (25). Such dietary patterns may contribute to cancer development through obesity, metabolic dysfunction, chronic low-grade inflammation, altered gut microbiota, exposure to carcinogenic compounds from processed meat and high-temperature cooking (25, 26), and high salt intake (27–29). However, these mechanisms provide background biological plausibility and were not directly tested in the cohort-level meta-analysis.

The EAT-Lancet diet may address several of these dietary risks by emphasizing plant-based foods and limiting red meat, processed foods, and added sugars. Nevertheless, the strength of evidence differs across cancer types, and the present findings should not be used to infer that the diet alone determines cancer risk. Cancer is multifactorial, and the observed associations may reflect both dietary exposure and correlated healthy behaviors.

The EAT-Lancet diet addresses the aforementioned shortcomings and offers a different approach. The EAT-Lancet diet is a predominantly plant-based dietary pattern. It recommends a daily intake of approximately 232 grams of whole grains (such as rice, wheat, or corn), 300 grams of vegetables, 200 grams of fruit, 125 grams of legumes (beans, lentils), and 50 grams of nuts. The diet also includes moderate amounts of unsaturated plant oils (40 grams), and allows for modest consumption of dairy foods (250 grams), fish (28 grams), and poultry (29 grams) per day. Red meat (beef, lamb, pork) is limited to about 14 grams per day, while eggs are restricted to 13 grams per day (roughly 1.5 eggs per week). The diet strongly limits added sugars (no more than 31 grams per day) and starchy vegetables (50 grams per day), and recommends minimizing the intake of highly processed foods and animal-source foods overall (9).

Mechanistically, the cancer-protective effects of the EAT-Lancet diet are likely multifactorial. First, a plant-forward dietary pattern increases fiber intake from whole grains, legumes, fruits, and vegetables. Fiber fermentation by gut microbiota produces short-chain fatty acids, especially butyrate, which may support intestinal barrier integrity, regulate inflammatory signaling, promote epithelial differentiation, and inhibit abnormal proliferation in colonic epithelial cells (30, 31). This pathway is consistent with EAT-Lancet-related cohort evidence for colorectal cancer, although our pooled colorectal cancer subgroup did not reach statistical significance (32). Second, fruits, vegetables, whole grains, nuts, and legumes provide phytochemicals, polyphenols, vitamins, and minerals that may reduce oxidative stress and influence cell-signaling pathways involved in carcinogenesis (30) (33, 34). Third, the EAT-Lancet pattern limits red and processed meats, which may reduce exposure to heme iron, N-nitroso compounds, and carcinogens generated during high-temperature cooking (26). Its emphasis on lower salt intake may also be relevant to gastric cancer risk, as supported by meta-analytic and prospective evidence on salt and salted foods (27–29). Fourth, limiting added sugars and energy-dense ultra-processed foods may help weight control and insulin sensitivity, thereby reducing obesity-related metabolic dysfunction and chronic low-grade inflammation (35). Systemic immune-inflammation index and neutrophil-to-lymphocyte ratio may partly mediate mortality associations (36). These pathways may operate differently across cancer sites: colorectal cancer may be more closely linked to fiber and gut microbiota, gastric cancer to salt-related pathways, and lung cancer to inflammatory and oxidative pathways that overlap with smoking-related risk. Overall, these mechanisms fit the direction of the overall association observed in our study, but they cannot establish causality because the included cohorts did not directly test these biological mechanisms.

Diet may influence different cancer types through different pathways. In the present subgroup analyses, lung cancer showed the clearest association with higher EAT-Lancet diet adherence scores. This finding may be related to higher intake of antioxidant- and fiber-rich plant foods and lower consumption of red and processed meats. Because smoking is the dominant risk factor for lung cancer, we checked the smoking adjustment in the two included lung cancer cohorts. Liu et al. adjusted for smoking status and pack-years of smoking in the UK Biobank analysis, whereas Xiao et al. adjusted for smoking status and daily cigarette consumption in the PLCO analysis (11) (13). Nevertheless, smoking variables were not harmonized across cohorts, and details such as smoking duration, cessation timing, passive smoking exposure, and changes after baseline were not consistently available. Therefore, residual confounding by smoking cannot be ruled out, and the lung cancer finding should be interpreted cautiously.

Updated WCRF/CUP Global evidence indicates that adulthood dietary and lifestyle patterns can influence breast cancer risk (37), and some studies also suggest that dietary factors may be relevant to prostate cancer outcomes (38). However, evidence remains mixed across specific dietary definitions, populations, and exposure categories (17) (39, 40). For breast and prostate cancer, no statistically significant association was observed in this meta-analysis. The null findings may reflect the limited number of eligible studies, heterogeneous definitions of the EAT-Lancet diet, different cutoffs for high and low adherence, and variation in dietary assessment instruments such as FFQs and 24-hour dietary recalls. Breast and prostate cancer risk is also shaped by hormonal, reproductive, genetic, metabolic, and lifestyle factors (37) (41–43), and adjustment for these variables differed across cohorts. These issues may have diluted the observable association between EAT-Lancet diet adherence and site-specific cancer risk.

This study has several strengths. First, it synthesized 15 moderate-to-high quality cohort studies, including large-scale cohorts such as the UK Biobank, PLCO, NHS/HPFS, and other population-based cohorts. Second, the long follow-up duration across studies allowed assessment of long-term cancer outcomes. Third, we updated the evidence base by including recent eligible studies and summarized how adherence scoring systems differed across cohorts. Fourth, subgroup analyses by cancer type and adherence level provided more detailed insight into the heterogeneity of the association between EAT-Lancet diet adherence and cancer outcomes. Finally, the inclusion of studies from multiple countries (e.g., the UK, US, Spain, Singapore) enhances the generalizability of the findings to diverse populations with varying socioeconomic and dietary backgrounds.

This study also has several limitations. First, substantial heterogeneity was observed for overall cancer incidence and moderate heterogeneity for cancer mortality. This heterogeneity likely reflects differences in cancer type, population characteristics, dietary habits, adherence scoring systems, increment units, exposure cutoffs, and dietary assessment instruments. Although subgroup and sensitivity analyses were conducted, the number of studies in several subgroups was small, limiting statistical power. Second, adherence to the EAT-Lancet diet was not operationalized uniformly across cohorts. Different studies used different scoring systems, dietary tools, increment units, and categorical adherence definitions, which reduces comparability. Because the increment units could not be harmonized, the pooled HRs should be interpreted as the overall direction and strength of the association. Third, most included studies relied on self-reported dietary data, which are prone to recall and measurement error. Fourth, most cohorts were from Europe and North America, which may limit generalizability to populations with different dietary patterns, food systems, socioeconomic contexts, and genetic backgrounds. Fifth, publication bias assessment suggested possible small-study effects for cancer incidence, although this result should be interpreted cautiously because fewer than 10 studies were included. Sixth, although included studies were generally rated as moderate to high quality and used adjusted estimates, residual confounding remains possible, particularly from smoking, alcohol consumption, physical activity, cancer screening, and other health-related behaviors. Finally, grey literature and records without full-text data were not included, which may introduce selection bias.

## Conclusion

This meta-analysis evaluated the association between adherence to the EAT-Lancet diet and cancer incidence and mortality in adults. Higher EAT-Lancet diet adherence scores were associated with lower overall cancer incidence and mortality, and the clearest site-specific association was observed for lung cancer. No statistically significant associations were found for breast, prostate, or colorectal cancer incidence. Because of heterogeneity, differences in adherence measurement, limited subgroup data, and possible residual confounding, these findings should be interpreted cautiously. Further large, prospective, multicenter studies using harmonized dietary scoring and comprehensive confounder adjustment are needed.

## Data Availability

The original contributions presented in the study are included in the article/**Supplementary Material**. Further inquiries can be directed to the corresponding author.

## Supplementary material

Supplementary Figure 1 Sensitivity analysis for the association between EAT-Lancet diet adherence and cancer incidence.

Supplementary Figure 2 Funnel plot for the association between EAT-Lancet diet adherence and overall cancer incidence.

Supplementary Figure 3 Sensitivity analysis for the association between EAT-Lancet diet adherence and overall cancer mortality.

Supplementary Figure 4 Funnel plot for the association between EAT-Lancet diet adherence and overall cancer mortality.

Supplementary Table 1 Additional information about studies included in the meta–analysis.

Supplementary Table 2 Quality assessment of the cohort studies included.
