# Supplementary figures and images for: Association between EAT-Lancet diet adherence and cancer incidence/mortality: a systematic review and meta-analysis
Source: Front Oncol. 2026 Jun 1;16:1823812. doi: 10.3389/fonc.2026.1823812 (PMC13265285; doi:10.3389/fonc.2026.1823812)

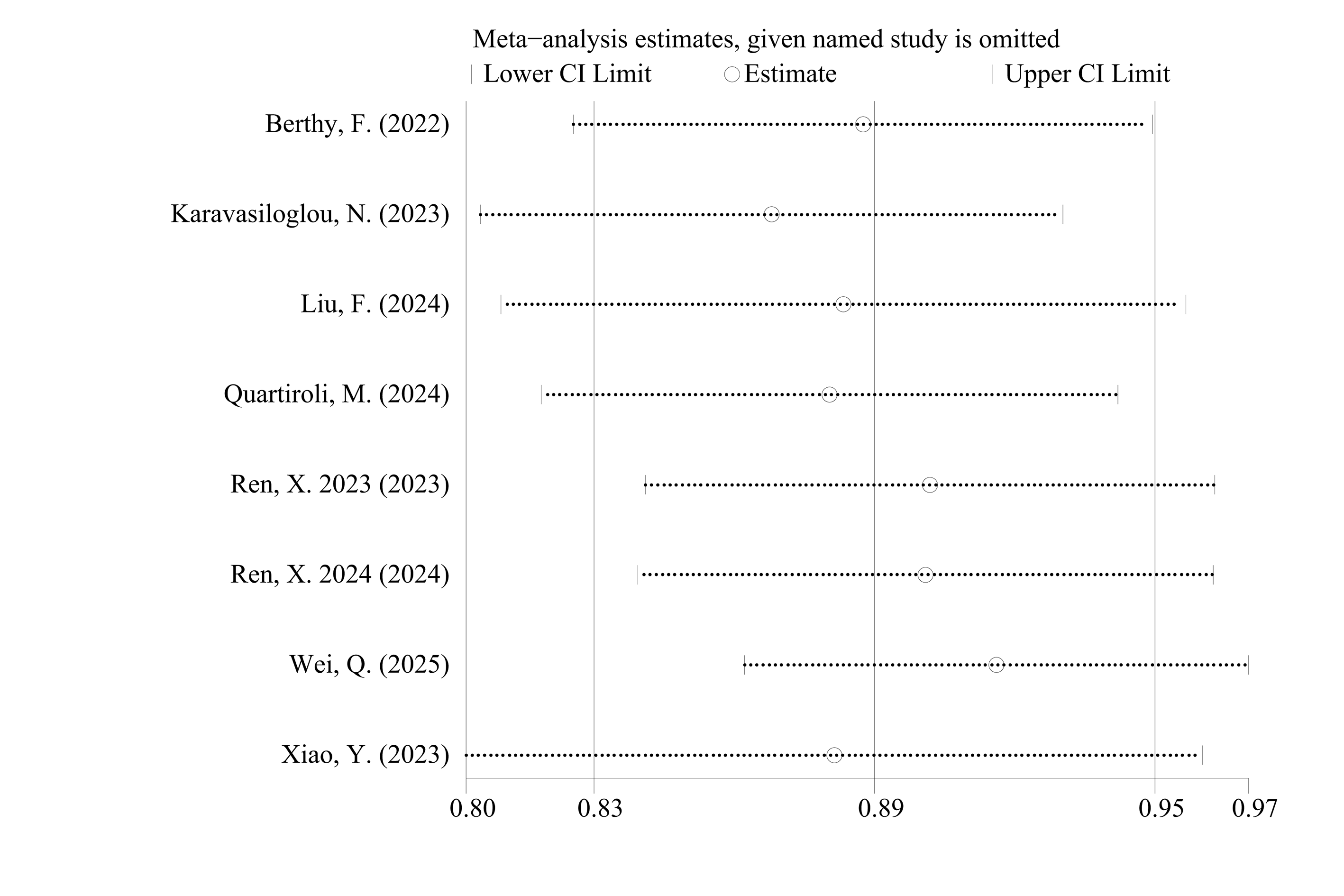

Supplement: Supplementary Figure 1 — Sensitivity analysis for the association between EAT-Lancet diet adherence and cancer incidence. [file Image1.tif]

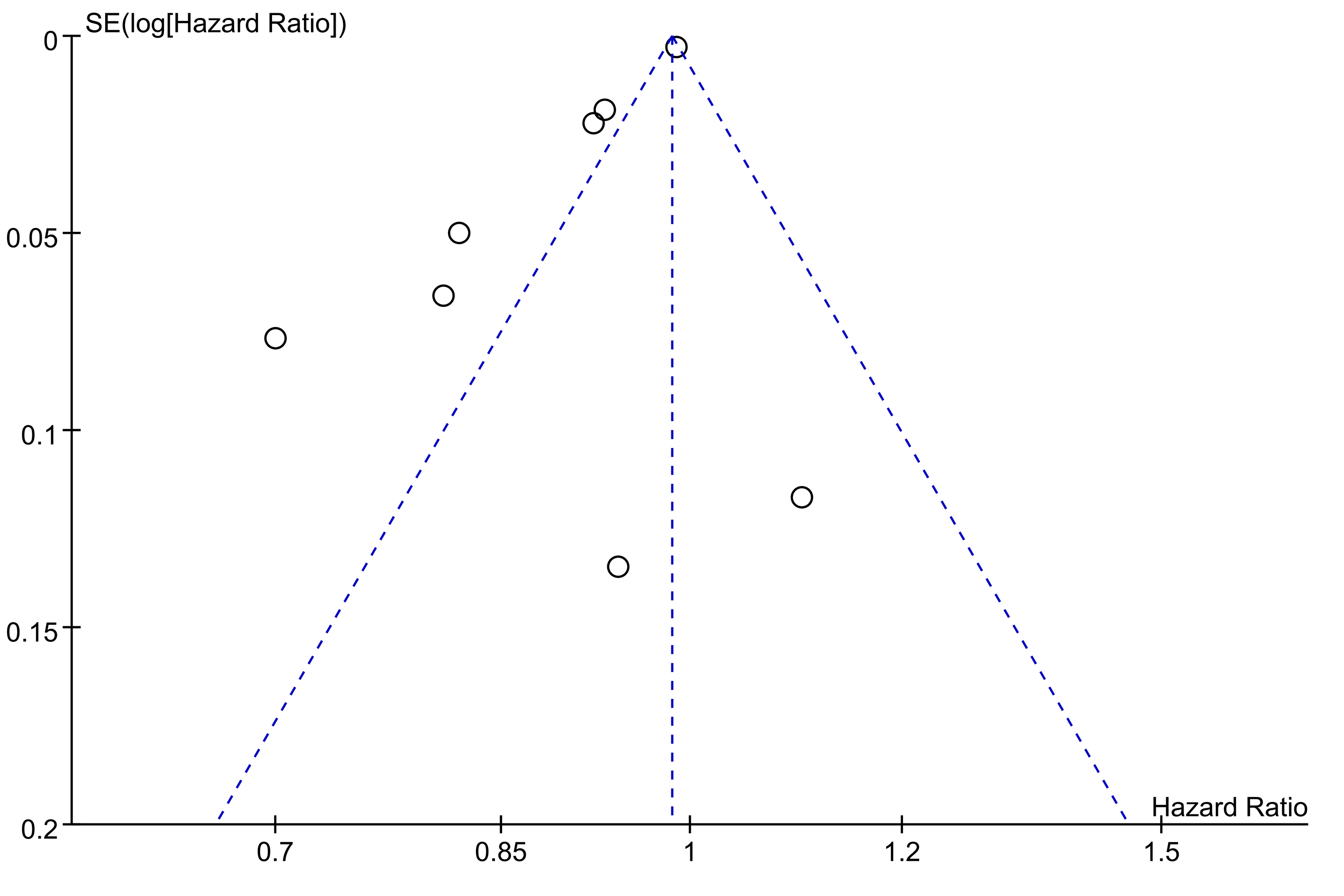

Supplement: Supplementary Figure 2 — Funnel plot for the association between EAT-Lancet diet adherence and overall cancer incidence. [file Image2.tif]

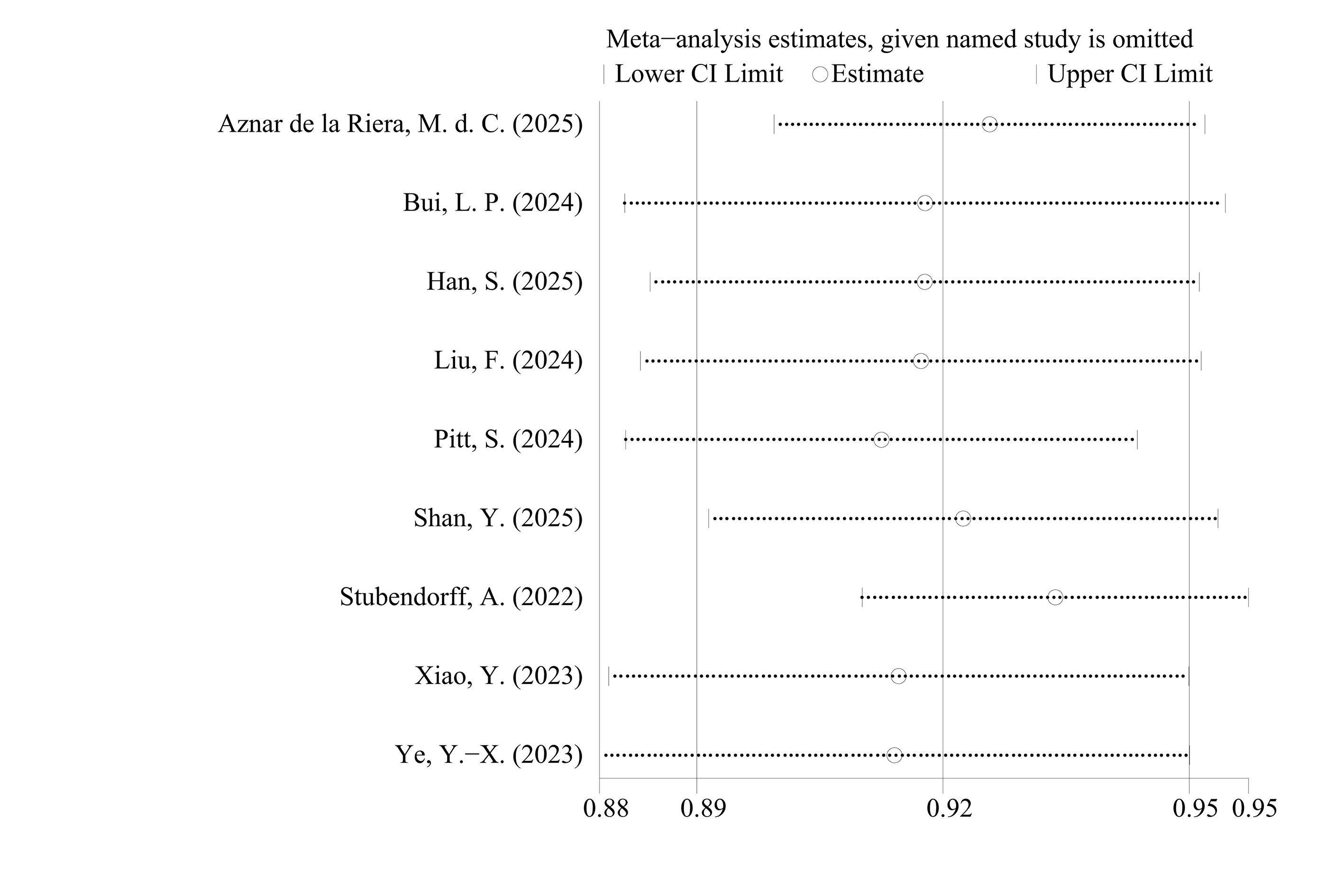

Supplement: Supplementary Figure 3 — Sensitivity analysis for the association between EAT-Lancet diet adherence and overall cancer mortality. [file Image3.tif]

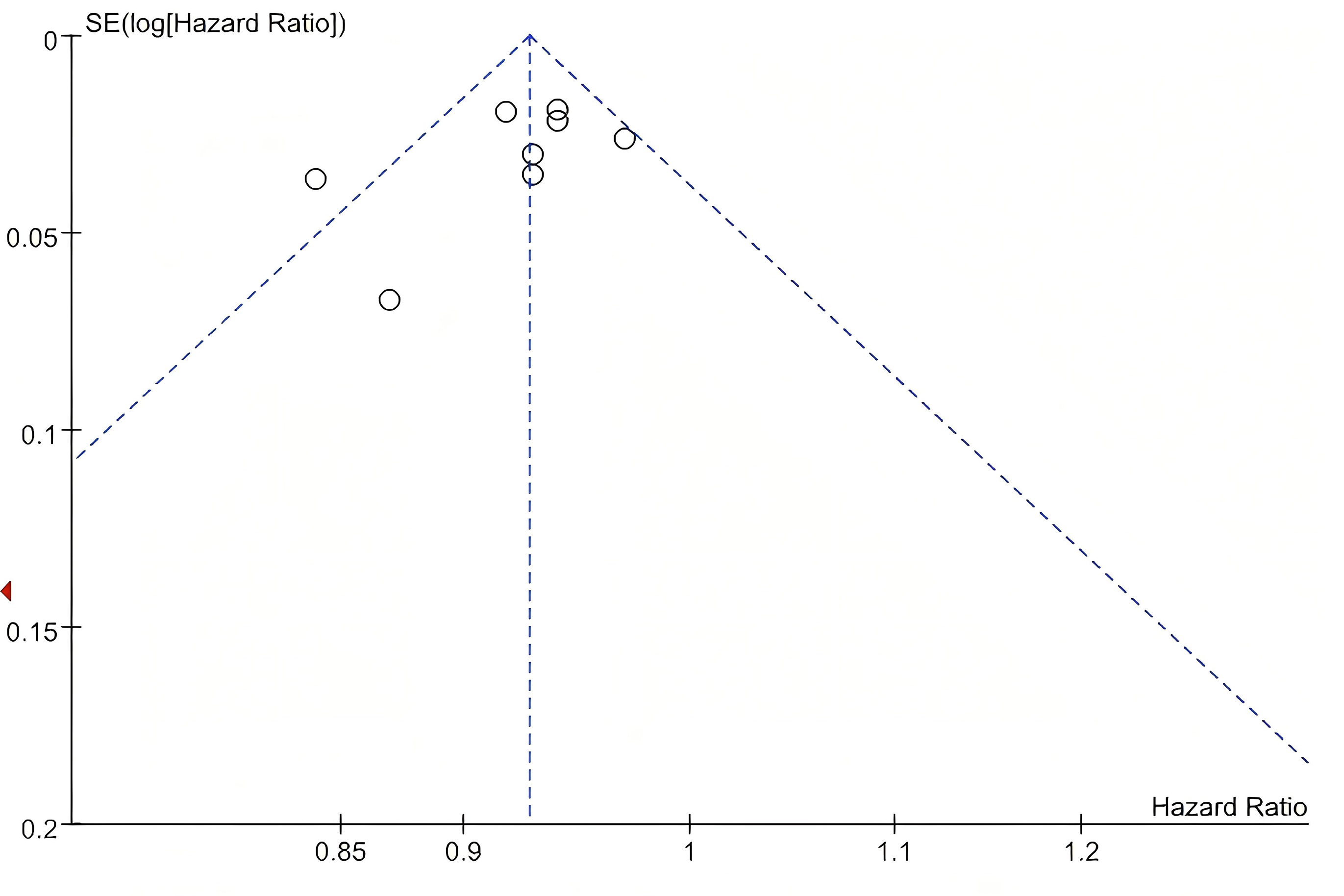

Supplement: Supplementary Figure 4 — Funnel plot for the association between EAT-Lancet diet adherence and overall cancer mortality. [file Image4.tif]
