# Supplementary material for: Association between EAT-Lancet diet adherence and cancer incidence/mortality: a systematic review and meta-analysis
Source: Front Oncol. 2026 Jun 1;16:1823812. doi: 10.3389/fonc.2026.1823812 (PMC13265285; doi:10.3389/fonc.2026.1823812)
Supplement: Supplementary Table 1 — Additional information about studies included in the meta–analysis. [file Table1.docx]

**Supplementary Table 1. Additional information about studies included in the meta–analysis.**

| Author | Year | Age (years) | EAT–Lancet Diet Adherence Grouping Criteria | EAT–Lancet Diet Scoring System Details |
| --- | --- | --- | --- | --- |
| Karavasiloglou, N. | 2023 | 56.50±8.08 | 3 categories (low: 0–4, moderate: 5–7, high: 8–11) | EAT–Lancet reference diet score; 7 dietary components; 1 point for meeting recommendations, 0 otherwise; total 0–11 points |
| Liu, F. | 2024 | 58.09±8.03 | 5 groups (Q1: ≤8, Q2: 9, Q3: 10, Q4: 11, Q5: ≥12) | EAT–Lancet Diet Score; 0–1 point per component; total 0–14 points |
| Ren, X. | 2023 | 65.52±5.73 | 4 quartiles (Q1: ≤18, Q2: 19–21, Q3: 22–24, Q4: ≥25) | ELD Score; 14 dietary components; 0–3 points/component; total 0–42 points |
| Xiao, Y. | 2023 | 65.5±5.70 | 4 quartiles (Q1: 4–9, Q2: 10, Q3: 11, Q4: 12–13) | ELD Score (adapted for US/European diets); 14 dietary components; 1 point for meeting recommendations, 0 otherwise; total 0–14 points |
| Wei, Q. | 2025 | 65.53±5.73 | 4 quartiles (Q1: ≤18, Q2: 19–21, Q3: 22–24, Q4: ≥25) | ELD Score; 14 dietary components; 0–3 points/component; total 0–42 points |
| Ren, X. | 2024 | 65.5±5.70 | 4 quartiles (Q1: 6–17, Q2: 18–21, Q3: 22–23, Q4: 24–40) | ELD Score; 14 dietary components; 0–3 points/component; total 0–42 points |
| Berthy, F. | 2022 | 51.0±10.2 | 5 quintiles (Q1–Q5) | ELD–I; 13 dietary components; continuous score: -162 to 332 points; mean 45.4±25.6 points |
| Quartiroli, M. | 2024 | 35–69 | Tertiles (T1: 4–9, T2: 10, T3: 11–12) | EAT-Lancet score; 14 dietary components, 0 or 1 point per component; excluded soy foods and nuts due to FFQ limitations; total score 0–12 points |
| Pitt, S. | 2024 | 21-69 | 4 groups (Low: ≤6, Low–middle: 7–8, High–middle: 9–10, High: ≥11) | EAT–Lancet Adherence Index: 14 dietary components; 0–1 point/component; total 0–14 points |
| Han, S. | 2025 | 47.02±17.01 | 4 quartiles (Q1: ≤40, Q2: 40–50, Q3: 50–61, Q4: ≥61) | **Planetary Health Diet Index for the United States**; 16 dietary components; total 0–150 points |
| Aznar de la Riera, M. d. C. | 2025 | 47.5±16.7 | Tertiles (T1–T3) | PHDI; 15 dietary groups; total 0–140 points |
| Stubbendorff, A. | 2022 | 45-73 | 5 groups (≤13, 14–16, 17–19, 20–22, ≥23) | ELD–I; 14 dietary components; 0–3 points/component; total 0–42 points |
| Bui, L. P. | 2024 | NHS1: 30-55, NHS2: 25-42,  HPFS: 40-75 | 5 quintiles (Q1–Q5) | PHDI; 15 dietary groups; total 0–140 points |
| Shan, Y. | 2025 | 21-69 | 5 quintiles (Q1: <71.2, Q2: 71.2–78.2, Q3: 78.2–84.4, Q4: 84.4–91.4, Q5: ≥91.4) | PHDI; 15 dietary groups; 0 points for non–adherence, 140 points for full adherence; total: 0–140 points |
| Ye, Y.-X. | 2023 | 56±17.90 | 5 quintiles (Q1: 13–47; Q2: 47–53; Q3: 53–58; Q4: 58–63; Q5: 63–95) | PHD Score; 14 dietary components; 0–10 points/component; total 0–140 points |

PHD, Planetary Health Diet; PHDI, Planetary Health Diet Index; ELD-I, EAT-Lancet Diet-Index; ELD, EAT-Lancet Diet.
