# Supplementary material for: Association between EAT-Lancet diet adherence and cancer incidence/mortality: a systematic review and meta-analysis
Source: Front Oncol. 2026 Jun 1;16:1823812. doi: 10.3389/fonc.2026.1823812 (PMC13265285; doi:10.3389/fonc.2026.1823812)
Supplement: Supplementary Table 2 — Quality assessment of the cohort studies included. [file Table2.doc]

**Supplementary Table 2. Quality assessment of the cohort studies included.**

| Author, year | **Selection (Out of 4)** | | | | **Comparability**  **(Out of 2)** | **Outcomes (Out of 3)** | | | **Total**  **(Out of 9)** |
| --- | --- | --- | --- | --- | --- | --- | --- | --- | --- |
|  | Representativeness of exposed cohort | Selection of non exposed cohort | Ascertainment  of exposure | Outcome not present at the start of the study |  | Assessment of outcomes | Length of follow-up | Adequacy of follow up of cohorts |  |
| Karavasiloglou,N., 2023 | 1 | 1 | 0 | 1 | 2 | 1 | 1 | 1 | 8 |
| Liu, F., 2024 | 0 | 1 | 1 | 1 | 2 | 1 | 1 | 1 | 8 |
| Ren, X., 2023 | 1 | 1 | 1 | 1 | 2 | 1 | 1 | 1 | 9 |
| Xiao, Y., 2023 | 1 | 1 | 1 | 1 | 2 | 1 | 1 | 1 | 9 |
| Wei, Q., 2025 | 1 | 1 | 1 | 1 | 2 | 1 | 1 | 1 | 9 |
| Ren, X., 2024 | 1 | 1 | 1 | 1 | 1 | 1 | 1 | 1 | 8 |
| Berthy, F., 2022 | 0 | 1 | 1 | 1 | 2 | 0 | 1 | 1 | 7 |
| Quartiroli, M., 2024 | 1 | 1 | 1 | 1 | 2 | 1 | 1 | 1 | 9 |
| Pitt, S., 2024 | 1 | 1 | 1 | 0 | 2 | 1 | 1 | 0 | 7 |
| Han, S., 2025 | 1 | 1 | 1 | 0 | 2 | 1 | 1 | 1 | 8 |
| Aznar de la Riera, M. d. C., 2025 | 1 | 1 | 1 | 0 | 2 | 1 | 1 | 0 | 7 |
| Stubbendorff, A., 2022 | 1 | 1 | 1 | 1 | 2 | 1 | 1 | 1 | 9 |
| Bui, L. P., 2024 | 1 | 1 | 1 | 1 | 2 | 1 | 1 | 1 | 9 |
| Shan, Y., 2025 | 1 | 1 | 1 | 1 | 2 | 1 | 1 | 0 | 8 |
| Ye, Y.-X., 2023 | 1 | 1 | 1 | 0 | 2 | 1 | 1 | 0 | 7 |

The cohort studies were assessed by the Newcastle-Ottawa Quality Assessment Scale (NOS) checklist.
